# The Histopathological Spectrum of Liver Diseases in Medico-Legal Autopsies: Findings From a Tertiary Care Center in Bangladesh

**DOI:** 10.7759/cureus.105944

**Published:** 2026-03-26

**Authors:** Mahmudul Haque Badhan, Sharmistha Roy, Javed Imran, Md. Ashik Kamal Alvee

**Affiliations:** 1 Department of Pathology, Abdul Hamid Medical College, Kishoreganj, BGD; 2 Department of Biochemistry, National Institute of Burn and Plastic Surgery, Dhaka, BGD; 3 Department of Pathology, Dhaka Medical College Hospital, Dhaka, BGD

**Keywords:** bangladesh, cirrhosis, histopathology, liver disease, medico-legal autopsy, steatohepatitis

## Abstract

Background and objective

Liver diseases often remain clinically silent until they reach advanced stages, and medico-legal autopsies can uncover previously undiagnosed pathologies. This study aimed to characterize the histopathological spectrum of liver diseases in medico-legal autopsies from a tertiary care center in Bangladesh.

Methods

This descriptive cross-sectional study was conducted in the Department of Pathology, Dhaka Medical College, Bangladesh, between September 1, 2021, and February 28, 2022. All adult (≥ 18 years) liver specimens obtained from medico-legal autopsies during the study period were consecutively included (n = 200). Demographic information and documented causes of death were collected using a structured form. Representative liver tissues were processed for routine histopathology and evaluated by light microscopy; lesions were categorized into steatosis, steatohepatitis, chronic hepatitis, cirrhosis, congestion, abscess, and malignant lesions. The data were analyzed using SPSS Statistics v22.0 (IBM Corp., Armonk, NY); Fisher's exact test was applied, and a p-value < 0.05 was considered significant.

Results

The mean age of the patients was 38.5 ± 11.05 years; the majority were male (96%), and most came from rural areas (61.5%). “Prisoners in cell” was recorded as the leading cause of death (32%). The liver weight distribution did not differ significantly by sex (p = 0.447). Histopathology revealed no significant abnormalities in 25% of patients. The most common lesions were steatohepatitis (20%) and cirrhosis (20%), followed by hepatic malignancies (17%), which included primary tumors (6%) and secondary tumors (11%). Other findings included steatosis (10%), chronic hepatitis (5%), congestion (2%), and hepatic abscess (1%). The sex distribution of major histopathological findings was also not statistically significant (p = 0.135).

Conclusions

Medico-legal autopsies revealed a substantial burden of previously undetected liver disease, dominated by steatohepatitis, cirrhosis, and malignancy. Routine histopathological evaluation via medico-legal autopsies may help identify concealed patterns of liver disease in Bangladesh.

## Introduction

Liver diseases represent a significant global health burden, contributing to 4% of all deaths (one out of every 25 deaths worldwide) [1]. The liver, an organ vital for numerous metabolic, synthetic, and detoxification processes, is highly susceptible to a diverse array of insults, including metabolic disturbances, toxic exposures, microbial infections, and circulatory compromise [2,3]. Due to the substantial functional reserve of the liver, many chronic liver pathologies often remain clinically silent until advanced stages, leading to late diagnoses or incidental findings during postmortem examinations [4,5]. Understanding the histopathological spectrum of these diseases, particularly in medico-legal autopsies, is crucial for elucidating the underlying causes of death, identifying undiagnosed conditions, and informing public health strategies [2,3,6]. Specifically, medico-legal autopsies offer a unique opportunity to analyze organ pathologies comprehensively and ascertain the true cause of death, even in cases where the clinical history is limited or unclear [6-8].

The epidemiological profile of liver diseases varies significantly by geography and is influenced by local factors such as socioeconomic status, lifestyle, dietary habits, and the prevalence of regional infections [6,9]. In Bangladesh, a developing nation in South Asia, liver diseases are a leading cause of death [10]. The region faces a high prevalence of viral hepatitis, with hepatitis E being the primary cause of acute hepatitis and hepatitis B virus (HBV) predominantly driving chronic liver disease and hepatocellular carcinoma [11,12]. Furthermore, nonalcoholic fatty liver disease (NAFLD), including nonalcoholic steatohepatitis (NASH), is emerging as a significant and increasingly common chronic liver disease that often progresses to cirrhosis and hepatocellular carcinoma [13,14]. This rising prevalence of NAFLD and NASH challenges the idea that HBV is the leading cause of chronic hepatitis in Bangladesh, indicating a shifting landscape of liver pathology [14]. These conditions are closely linked to metabolic syndrome and insulin resistance, highlighting systemic metabolic dysfunction as a core pathophysiological driver [13].

Studies focusing on the histopathological spectrum of liver diseases in autopsy cases from tertiary care centers in various settings globally have offered important insights into patterns of disease prevalence and progression [2,3,7,8,15]. These investigations often reveal a large proportion of previously undiagnosed liver conditions, highlighting the diagnostic value of postmortem examinations [4,5]. For example, a previous study in Bangladesh described the demographic and virological characteristics of patients with liver cirrhosis, identifying HBV and HCV as frequent causes, with many patients unaware of their infection status [16]. Another study from Bangladesh reported that chronic liver disease was among the leading causes of death in a tertiary hospital setting [10]. Despite the considerable burden of liver diseases in Bangladesh, detailed data on the histopathological spectrum of liver diseases derived from medico-legal autopsies, especially from tertiary care centers, are still scarce. This knowledge gap limits a comprehensive understanding of the true prevalence, spectrum, and contributing factors of liver diseases in the population, which is crucial for designing targeted public health strategies and enhancing clinical practice.

This study aimed to characterize the histopathological spectrum of liver diseases in medico-legal autopsies conducted at a tertiary care center in Bangladesh. It also aimed to outline the demographic profile of the cases and assess sex-based differences in major histopathological findings, thereby offering a clearer perspective on the burden of previously undetected liver disease in this population.

## Materials and methods

Study design and setting

This descriptive cross-sectional study was conducted in the Department of Pathology, Dhaka Medical College, Bangladesh. The study was conducted over six months, from September 1, 2021, to February 28, 2022.

Study population

All liver specimens included in the histopathology section from medico-legal autopsy cases during the study period were eligible for inclusion, and a total of 200 liver specimens were included in the final analysis.

Sample size calculation

The minimum required sample size was calculated via the following formula:

\[ n = \frac{Z^2 pq}{d^2} \]

Assuming a prevalence (p) of pathological liver findings of 11.7% based on prior autopsy-based evidence from Bangladesh [17], a 95% confidence level (Z = 1.96), and a margin of error of 5%. This yielded a minimum sample size of 159 cases. To account for potential exclusions due to autolysis or inadequate samples, a greater number of cases were included, resulting in a final sample size of 200.

Sampling technique

A consecutive sampling technique was applied, whereby all eligible autopsy liver specimens received during the study period were consecutively included.

Inclusion and exclusion criteria

Autopsy cases of adult individuals (≥ 18 years) of both sexes were included, provided that permission was obtained from the appropriate legal authority and that informed consent was granted by the next of kin. Pediatric cases, exhumed bodies, and severely autolyzed liver specimens were excluded from the study.

Data collection

Relevant demographic information, including age, sex, and area of residence, as well as the documented cause of death, was collected from first-degree relatives. All the data were recorded via a prestructured data collection form designed specifically for this study.

Specimen collection and gross examination

Liver tissue samples were procured either as components of a comprehensive multiple viscera examination or as intact whole-organ specimens from medico-legal autopsies. Accompanying demographic data, such as age and sex, were recorded from the official autopsy documentation. A thorough gross examination of the liver samples was conducted, during which characteristics such as color, consistency, surface nodularity, and the presence of autolytic changes were meticulously noted. Liver tissue sampling was performed from representative areas, including both macroscopically normal and abnormal regions where applicable. In cases of focal lesions, targeted sampling was done from the lesion as well as the adjacent surrounding tissue to ensure comprehensive evaluation. When whole organs were available, the liver weight was recorded in accordance with standard autopsy protocols.

Tissue processing and staining

Tissue samples (approximately 2 × 2 × 2 cm) were fixed in 10 % neutral buffered formalin, processed via standard paraffin‑embedding techniques, and sectioned at 4-5 µm thickness via a rotary microtome [18]. Dewaxed sections were routinely stained with hematoxylin and eosin (H&E) for light microscopic evaluation [18]. Masson’s trichrome stain is used as a special stain to assess fibrosis; this stain imparts a blue color to collagen against a red cytoplasmic background, and highlights type I collagen within portal tracts and fibrous septa [19].

Histopathological evaluation and diagnostic criteria

Microscopic evaluation focused on architectural and cellular features using established diagnostic criteria from standard hepatopathology references. Histopathological assessment was performed using established standard diagnostic criteria based on recognized hepatopathology references; however, no formal numerical scoring system (such as the NAFLD Activity Score) was applied. Steatosis was diagnosed when ≥ 5 % of hepatocytes contained intracytoplasmic lipid vacuoles; in NAFLD, these vacuoles are typically macrovesicular [20,21]. Steatohepatitis is defined by the coexistence of steatosis with lobular inflammation and hepatocellular ballooning [20]. Ballooned hepatocytes are characterized by enlarged cells with pale cytoplasm and wispy eosinophilic strands, and their presence has prognostic significance [20]. The lobular inflammatory infiltrate comprises mixed lymphocytes and Kupffer cell aggregates [22]. Chronic hepatitis is diagnosed when portal tracts contain mild to moderate mononuclear (predominantly lymphocytic) inflammation with interface activity and lobular necroinflammatory changes [23].

Cirrhosis is identified by diffuse nodularity of the hepatic parenchyma; regenerative nodules are separated by fibrous septa containing chronic inflammatory infiltrates and ductular reactions, reflecting distortion of the normal hepatic architecture [24,25]. Hepatic malignancies were categorized as primary (hepatocellular carcinoma or cholangiocarcinoma) or secondary (metastatic) based on histomorphological features and established diagnostic guidelines. Hepatocellular carcinoma accounts for 80-85 % of primary hepatic malignancies, whereas cholangiocarcinoma accounts for 15-20 % [26,27]. Any ambiguous findings were resolved by consensus between two experienced pathologists. Special stains (Masson’s trichrome) were selectively applied to assess fibrosis. Immunohistochemistry was not performed routinely due to resource limitations and was reserved only for selected cases where morphological diagnosis was inconclusive. All histopathological slides were independently reviewed by two experienced pathologists, and any discrepancies were resolved through consensus to minimize interobserver variability.

Statistical analysis

The data were entered, cleaned, and analyzed via SPSS Statistics version 22.0 (IBM Corp., Armonk, NY). Categorical variables are expressed as frequencies and percentages, whereas continuous variables are summarized as the means ± standard deviations (SD). The Fisher's exact test was applied to examine associations between sex and categorical study variables, including liver weight categories and major histopathological diagnoses. A p-value of less than 0.05 was considered statistically significant.

Ethical considerations

Ethical approval for the study was obtained from the Ethical Review Committee of Dhaka Medical College (Memo No: ERC-DMC/ECC/2021/236). Authorization for the use of medico-legal autopsy specimens was obtained from the appropriate legal authorities in accordance with national regulations. Written informed consent for the use of autopsy tissues and related information for research purposes was obtained from the next of kin. All personal and clinical information was kept strictly confidential, and no identifying details were disclosed at any stage of the study.

## Results

The mean age of the cohort was 38.5 ± 11.05 years. Most of the autopsied patients were male (n = 192; 96%), while females accounted for a small proportion (n = 8; 4%). The majority of cases were from rural areas (n = 123, 61.5%), with the remaining 38.5% from urban areas (Table 1).

**Table 1:** Baseline characteristics of the autopsy cohort (n = 200) SD: standard deviation

Variables	Category	Frequency (%)
Age (years)	Mean ± SD	38.5 ± 11.05
Sex	Male	192 (96)
	Female	8 (4)
Residence	Rural	123 (61.5)
	Urban	77 (38.5)

With respect to the causes of death, the most frequent category was “prisoners in cells,” accounting for 64 cases (32%). Natural death was recorded in 47 patients (23.5%), whereas no remarkable cause of death was identified in 53 patients (26.5%). Previously diagnosed malignancies were identified as the cause of death in 18 patients (9%). Other causes included police custody (2%), alcohol poisoning (2%), road traffic accidents (1.5%), head injuries (1%), burns (1%), drowning (1%), and hanging (0.5%) (Table 2).

**Table 2:** Distribution of cases according to cause of death (n = 200)

Cause of death	Frequency (%)
Prisoners in cell	64 (32)
Natural death	47 (23.5)
Previously diagnosed malignancy	18 (9)
Police custody	4 (2)
Road traffic accident	3 (1.5)
Burns	2 (1)
Alcohol poisoning	4 (2)
Drowning	2 (1)
Hanging	1 (0.5)
Head injury	2 (1)
No remarkable cause identified	53 (26.5)

Gross examination revealed that liver weight most commonly ranged between 1500 and 2500 g in both males (53.6%) and females (50%). A liver weight between 1000 and 1500 g was observed in 42.7% of males and 37.5% of females (p = 0.447). The distribution of liver weight categories was not significantly associated with sex (Table 3).

**Table 3:** Liver weight distribution by sex (n = 200) Fisher’s exact test was performed

Liver weight (grams)	Male, n (%)	Female, n (%)	P-value
<1000	5 (2.6)	1 (12.5)	0.447
1000–1500	82 (42.7)	3 (37.5)	
1500–2500	103 (53.6)	4 (50)	
>2500	2 (1)	0 (0)	

Histopathological examination revealed that 50 patients (25%) had no remarkable liver pathology. Among the abnormal findings, steatohepatitis and liver cirrhosis were the most common lesions, each identified in 40 patients (20%). Hepatic malignancies were observed in 34 patients (17%), comprising 12 patients (6%) with primary hepatic malignancies and 22 patients (11%) with secondary hepatic malignancies. Other histopathological findings included steatosis in 20 patients (10%), chronic hepatitis in 10 patients (5%), congestion in four patients (2%), and hepatic abscess in two patients (1%) (Table 4).

**Table 4:** Histopathological spectrum of liver lesions in autopsies (n = 200)

Histopathological findings	Frequency (%)
No remarkable pathology	50 (25)
Steatohepatitis	40 (20)
Liver cirrhosis	40 (20)
Hepatic malignancies (total)	34 (17)
Hepatic primaries	12 (6)
Hepatic secondaries	22 (11)
Steatosis	20 (10)
Chronic hepatitis	10 (5)
Congestion	4 (2)
Hepatic abscess	2 (1)

On sex-wise analysis of major histopathological findings, steatohepatitis (20.8%) and liver cirrhosis (20.4%) were the most common lesions among males, followed by hepatic malignancies (16.6%). In contrast, the majority of female patients had no remarkable pathology (62.5%), whereas hepatic malignancy and cirrhosis were identified in 25% and 12.5% of female patients, respectively. Overall, there was no statistically significant association between sex and the major histopathological findings (p = 0.135) (Table 5).

**Table 5:** Major histopathological findings by sex (n = 200) Fisher's exact test was performed

Histopathological findings	Male, n (%)	Female, n (%)	P-value
No remarkable pathology	45 (23.4)	5 (62.5)	0.135
Steatohepatitis	40 (20.8)	0 (0)	
Liver cirrhosis	39 (20.4)	1 (12.5)	
Hepatic malignancies	32 (16.6)	2 (25)	
Steatosis	26 (13.6)	0 (0)	
Chronic hepatitis	10 (5.2)	0 (0)	

## Discussion

This study aimed to characterize the histopathological spectrum of liver diseases in medico-legal autopsies at a tertiary care center in Bangladesh. We demonstrated that a substantial proportion of autopsied livers harbored previously undiagnosed pathological changes, with steatohepatitis and liver cirrhosis being the most frequent findings, followed by hepatic malignancies, whereas one quarter of the cases had no remarkable histopathology, highlighting the silent burden of liver disease in this population. The study population predominantly comprised young to middle-aged adults, with an overwhelming male predominance and a significant proportion from rural areas. These demographic characteristics are consistent with those of typical medico-legal autopsy cohorts in many developing countries, often reflecting patterns of trauma, violence, and sudden unexpected deaths that disproportionately affect younger adult males [6]. The high representation from rural areas also aligns with the broader demographic distribution in Bangladesh.

Notably, one-fourth of the patients had no remarkable liver pathology, underscoring the liver's remarkable resilience and functional reserve, where early or mild changes might not manifest clinically or histopathologically at the time of death [4]. Among the abnormal findings, steatohepatitis and liver cirrhosis were the most common lesions, each identified in 20% of the cases. The prevalence of steatohepatitis highlights the increasing global burden of NAFLD and its more severe form, NASH, even in populations not traditionally associated with Western lifestyles [3]. This aligns with global trends showing that NAFLD is a rapidly emerging cause of chronic liver disease and a significant public health concern [28]. The finding of liver cirrhosis in an equally high proportion (20%) is also consistent with the established burden of chronic liver disease in Bangladesh, which is recognized as a leading cause of death in the region [10]. Common etiologies for cirrhosis in this context may include chronic viral hepatitis (hepatitis B and C), alcohol-related liver disease, and NASH; however, this study could not establish etiological associations due to the lack of clinical and serological data [28].

Hepatic malignancies constituted a substantial proportion of all abnormal findings, with secondary malignancies being more prevalent than primary hepatic malignancies. The high proportion of secondary malignancies may suggest that the liver is a frequent site for metastatic spread from other primary cancer sites in this population, which could be due to the late diagnosis of primary cancers or specific cancer prevalence patterns in Bangladesh [7]. The 6% prevalence of primary hepatic malignancies, predominantly hepatocellular carcinoma, is a serious concern, especially given that hepatitis B virus infection is a known major risk factor for hepatocellular carcinoma in Bangladesh; however, etiological attribution could not be confirmed in this study due to the absence of clinical and serological data [28]. Other significant histopathological findings included steatosis, chronic hepatitis, congestion, and hepatic abscess. Steatosis often represents the initial stage of NAFLD and can progress to steatohepatitis, emphasizing the continuum of fatty liver disease [3]. Chronic hepatitis, which is often associated with viral infections, is characterized by ongoing inflammatory liver damage that can lead to fibrosis and cirrhosis; however, specific etiologies could not be determined in this study [28].

The sex-wise analysis in this study revealed that steatohepatitis and liver cirrhosis were the most prevalent histopathological findings among males, followed by hepatic malignancies. This pattern of a relatively high prevalence of chronic and advanced liver pathologies in males is frequently observed in the global literature and is often attributed to sex-specific differences in exposure to risk factors such as alcohol consumption, occupational hazards, and dietary habits [6,29]. Conversely, the majority of female patients (62.5%) had no remarkable pathology. Despite these observed prevalence differences, the absence of a statistically significant association between sex and major histopathological findings implies that, once initiated, the fundamental pathological processes underlying these liver diseases may not differ fundamentally between sexes in this specific medico-legal autopsy cohort, which aligns with prior studies [8]. This finding aligns with broader autopsy findings where the liver's response to various insults can exhibit similar morphological changes irrespective of sex [5]. The limited number of female cases (4% of the total) might also influence the statistical power to detect subtle sex-specific differences, a common limitation in forensic studies [6].

The causes of death in this study, with “prisoners in cells” being the most frequent category, followed by “natural death” and “no remarkable cause of death identified,” highlight the complex nature of medico-legal investigations. The significant number of cases with “no remarkable cause of death identified” underscores the value of thorough histopathological examination, as silent liver pathologies could contribute to or provide important insights into the circumstances leading to death, even if not the direct cause [4,30]. This study, like others in similar settings, demonstrates the critical role of pathological examination of the liver in medico-legal autopsies, not only for determining the cause of death but also for understanding the demographic and pathological patterns of diseases that might otherwise remain undiagnosed [6].

Limitations

This study has several important limitations. As a single-center, tertiary hospital-based medico-legal autopsy study using consecutive sampling, there is a potential for selection bias, as the study population may not be fully representative of the general population of Bangladesh. This may limit the generalizability of the findings. In addition, detailed clinical history, laboratory investigations, imaging findings, alcohol use, metabolic risk factors, and viral serological data were not consistently available, restricting accurate etiological classification of liver diseases and limiting clinicopathological correlation. Sampling was confined to representative tissue sections, and special stains and immunohistochemistry were not applied systematically, which may have resulted in potential misclassification of certain lesions. Furthermore, the very small number of female cases reduced the statistical power for meaningful sex-based comparisons. Additionally, representative histopathological images could not be included, which may limit the visual illustration of key findings. Future multicenter studies incorporating comprehensive clinical data, standardized diagnostic protocols, and advanced ancillary techniques are warranted to address these limitations.

## Conclusions

Our study, based on the histopathological examination of medico-legal autopsies, revealed a considerable burden of previously undiagnosed liver diseases, with steatohepatitis, cirrhosis, and hepatic malignancies being the most frequent findings. These results highlight the silent nature of liver pathology in the population and emphasize the value of routine liver histopathology in medico-legal autopsies. Larger multicenter studies are needed to better define population-level patterns.
